# Novel ML-Based Algorithm for Detecting Seizures from Single-Channel EEG

**DOI:** 10.3390/neurosci5010004

**Published:** 2024-02-29

**Authors:** Yazan M. Dweiri, Taqwa K. Al-Omary

**Affiliations:** Department of Biomedical Engineering, Faculty of Engineering, Jordan University of Science and Technology, P.O. Box 3030, Irbid 22110, Jordan

**Keywords:** seizure classification, portable epilepsy monitoring, machine learning

## Abstract

There is a need for seizure classification based on EEG signals that can be implemented with a portable device for in-home continuous minoring of epilepsy. In this study, we developed a novel machine learning algorithm for seizure detection suitable for wearable systems. Extreme gradient boosting (XGBoost) was implemented to classify seizures from single-channel EEG obtained from an open-source CHB-MIT database. The results of classifying 1-s EEG segments are shown to be sufficient to obtain the information needed for seizure detection and achieve a high seizure sensitivity of up to 89% with low computational cost. This algorithm can be impeded in single-channel EEG systems that use in- or around-the-ear electrodes for continuous seizure monitoring at home.

## 1. Introduction

Epilepsy is one of the brain diseases that is gaining attention due to its spread and the extent of its impact on the daily lives of patients. According to the WHO, by 2031, the world is aiming to increase countries’ coverage of epilepsy services to 50% coverage in 2021. The appropriate diagnosis and treatment of people with epilepsy is estimated to allow 70% of these patients to have seizure-free lives.

The diagnosis of epilepsy relies on neurological examination and observations of symptoms. The investigation of the EEG is assigned as the confirmatory tool [1], besides examining some biomarkers obtained from ECG and sleep studies. Brain imaging using CT and MRI is also utilized in the diagnosis and assessment of epilepsy and seizures [2].

Epilepsy is managed pharmaceutically with customized anti-seizure medications. Different medications with different doses are suggested to patients according to the type of epilepsy, type of seizures, the frequency of its occurrence, age, sex, health condition, and other factors [3]. Recording seizure information is crucial for the assessment of medication and management of epilepsy. Alternatives for refractory epilepsy include resective surgery and diet therapy [4].

Some epileptic patients experience seizures day and night, whereas others suffer seizures only during daytime or only during night-time (~20%) [5,6]. Comparative studies between simultaneous EEG recordings and patients-counting of seizures have shown that self-reporting is ineffective as it leaves more than 50% of seizures unrevealed [7]. The ratio rises to 85.8% for nocturnal (i.e., during sleep) seizures [8].

The unpredictable nature of seizures is a serious challenge to the patients themselves and the people around them, in addition to the inability to recognize some types of asymptomatic seizures, which can lead to serious complications that may ultimately lead to sudden unexpected death in epilepsy (SUDEP) [9,10].

Therefore, the need for developing a practical method of continuous seizure detection has emerged to address two major aspects: providing physicians with detailed records of the timing and frequency of seizures and their types [1], along with the need to offer the necessary care for the patient during or shortly after seizures.

Cumulative efforts have produced user-friendly wearable devices have been produced to enhance the management of epilepsy and seizures continuously at home. According to the type of sensor and type of input, there are various seizure-detection systems. Some systems have one input, and others include a combination of inputs. Examples of such devices that indirectly monitor seizures without recording EEG include smart-watches-based systems, alert bracelets, movement monitors based on camera or motion sensing, and breathing monitors.

It is critically noted that some sensors are limited to detecting not all but certain types of seizures. The strategy for selecting a monitor for epilepsy should consider the seizure type according to these capabilities [11].

EEG monitoring is considered the gold standard for seizure detection and prediction. It detects the excitatory and inhibitory post-synaptic potentials (EPSPs and IPSPs) [11]. Regular and prolonged EEG testing and sleep studies are essential to explore individual behavior and reactions to medicine [12]. Studies in epilepsy underline the significant role of routine EEG in establishing the medical plan and modifying it, if necessary, for optimum treatment [12,13]. This can be achieved by accurate differentiation between an epileptic seizure and a nonepileptic event [14] and by being able to suspect a seizure afterward [15]. A comprehensive recording, including wakefulness, drowsiness, and sleep, is preferred [16].

Other systems take advantage of the symptoms and disorders associated with seizures, such as involuntary movement that is identified by video monitoring, muscle activity (EMG), accelerometry (ACM), and mattress sensors. Biomarkers are also utilized in such systems as heart rate changes in ECG-based systems, sweating in electrodermal activity (EDA) systems, and cerebral blood flow recognized by cerebral oxygen saturation sensors [11,17]. Multimodal systems, like combining heart rate and movement, have been used for seizure detection [18].

An option for long-term EEG monitoring is intracranial monitoring (iEEG). Commercially available iEEG monitoring devices can provide therapeutic neurostimulation once the onset of a seizure is detected. Besides the invasive nature of this approach and its moderate data-acquisition capability, it has been reported to be restricted to unifocal and bifocal epilepsy [19]. Additionally, scalp EEG is superior to iEEG in detecting absence seizures [11] and has shown higher sensitivity in simultaneous recordings [20].

Other options are deep brain stimulation (DBS)-based devices. These have been shown to sense brain signals while delivering stimulation to treat symptoms of epilepsy characterized by partial-onset seizures, with or without secondary generalization, that are refractory to three or more antiepileptic medications.

Behind-the-ear sensors form a simple EEG monitoring approach that can detect focal [21,22] and generalized epileptic seizures [23]. They have been shown to provide real-time EEG monitoring and determine seizure frequency and severity with an overall sensitivity of 75% and an accuracy range from 70% to 90% [24].

The work toward achieving an automatic detection of seizures based on EEG signals relies on exploring the features that identify ongoing seizures and distinguish them from normal EEG. The standard practice in analyzing raw EEG signals involves feature extraction followed by optimum feature selection to construct the feature vector and then classification based on a threshold or model standards. The feature can be extracted from several domains, categorized into time, frequency, time–frequency, and non-linear domains such as empirical mode decomposition (EMD) and rational transform domains. Researchers have shown that a merged combination of features from different domains provides better performance in comparison with a single-domain feature vector [25,26].

Various feature-extraction methods and machine learning classifiers based on single and multiple EEG channels were explored with a minimum accuracy of 70%. This emphasizes the improvements and added values that ML approaches have provided to this scope [27].

In seizure detection, an AI model is usually trained on segments of EEG data to classify them into ictal, interictal, and normal signals. Several types of neural networks, including recurrent neural networks (RNNs), have shown great potential in sequential signal classification, such as the brain signal [28].

Combining different types of classifiers to detect epileptic seizures was explored like combining an ANN feed-forward backpropagation and an ML support vector machine (SVM) [29]. The benchmark database acquired from Bonn University [30] The classification results showed higher sensitivity in many of the considered cases than methods that relied on decision trees, EMD, and other types of ANNs individually. Acharya et al. developed a deep convolutional neural network (CNN) algorithm that was trained on single-channel EEG segments extracted from the original multichannel dataset from Bonn University [31]. Nevertheless, deep neural networks usually require large training data for the network to make correct decisions and highly accurate classifications afterward. They also require higher computational power and time to run the fully trained network.

Particle Swarm Optimization (PSO)-based ANN was evaluated for seizure classification of each patient separately (patient-specific). It has a sensitivity of 98% [32]. Another study by Satapathy et al. proposed a close concept using the PSO algorithm with radial basis function ANN; it has shown high accuracies with different datasets and using different features [33].

Other types of ANNs have been utilized in early seizure detection, like a weightless neural network (WNN) called WiSARD [34], which performs the classification based on the raw EEG data rather than the extracted features obtained after the initial analysis. The dataset was obtained from the European epilepsy database (EPILEPSIAE) [35]. Classification accuracy ranges between 72.56% and 99.93% was reportedwith an anticipation time range from 2 to 30 s.

Another study focused on the enhancement of the dataset itself, and the sensitivity reached was relatively high [36].

One of the studies that addressed single-channel EEG for the automatic detection of epileptic events used the databases of Bonn and CHB-MIT. It used a novel feature for classification: Kraskov entropy based on the Hilbert–Huang Transform (HHT). Although it performed well with the dataset from Bonn, it did poorly with the other [37]. Another study of single-channel EEG classification was conducted on mice rather than patients, where Bergstrom et al. developed an algorithm based on wavelet decomposition, which had previously used the total multi-channel brain signal. In their study, they introduced a modified algorithm based on the same approach to classify single-channel signals into interictal spikes, seizures (including absence-type), and other abnormal events with an accuracy of 87% [38].

As noted from this literature review, most of the published studies dealing with seizure classification focus primarily on the accuracy of classification, without considering the complexity of their proposed approaches and thus their potential for implementation within a portable system for epilepsy for in-home use. Some studies perform the classification using other bio signals besides the EEG, rather than relying solely on the EEG signal. Also, studies that focus on the number of channels are rare. Usually, they use the total number of channels in the utilized databases.

Gradient tree boosting is a form of tree ensemble boosting introduced by Friedman in 2001 based on the idea of decision trees and random forests [39]. It is a technique used for creating a strong classifier based on merging weak classifiers and taking advantage of their weaknesses in a way that helps improve the final classification model. The way the final model is built is based on iterative learning and training on the data starting with the base model and moving toward the robust model. Iterative optimization of training leads to progressively enhancing the model (Gradient Boosting).

XGBoost was developed by Chen and Guestrin in 2016 [40]. It follows the same principle as gradient tree boosting;but provides improved performance by managing the complexity of the trees through the use of various regularization approaches.

The speed and performance of XGBoost make it a widely used model in the fields of regression, classification, ranking, and user-defined prediction problems. Since it was first proposed, XGBoost has shown advanced performance on ML tasks and consistently outperforms other single-algorithm methods, along with very fast training.

Several studies have shown the feasibility of detecting seizures using single-channel EEG. Furthermore, reviews in epilepsy have concluded that features extracted from time and time–frequency domains are the most used in seizure detection methods [41]. Another review confirms the same conclusion and adds that the variance, energy, nonlinear energy, and Shannon entropy features extracted from raw EEG signals are the best features for distinguishing seizures, providing that the variance, energy, kurtosis, and line length all have low computational complexity [27].

The presented study aims to develop a novel ML algorithm for seizure detection according to wearable system requirements and specifications using an extreme gradient boosting (XGBoost) classifier, as it was indicated to perform highly on seizure-detection problems using different combinations of features and EEG databases [42,43]. The combination of an efficient classifier with a single-channel EEG dataset obtained from an appropriate cranial location and the use of a feature vector of low computational complexity will meet the requirements of wearable systems while maintaining high classification performance.

## 2. Materials and Methods

**Figure 1:**
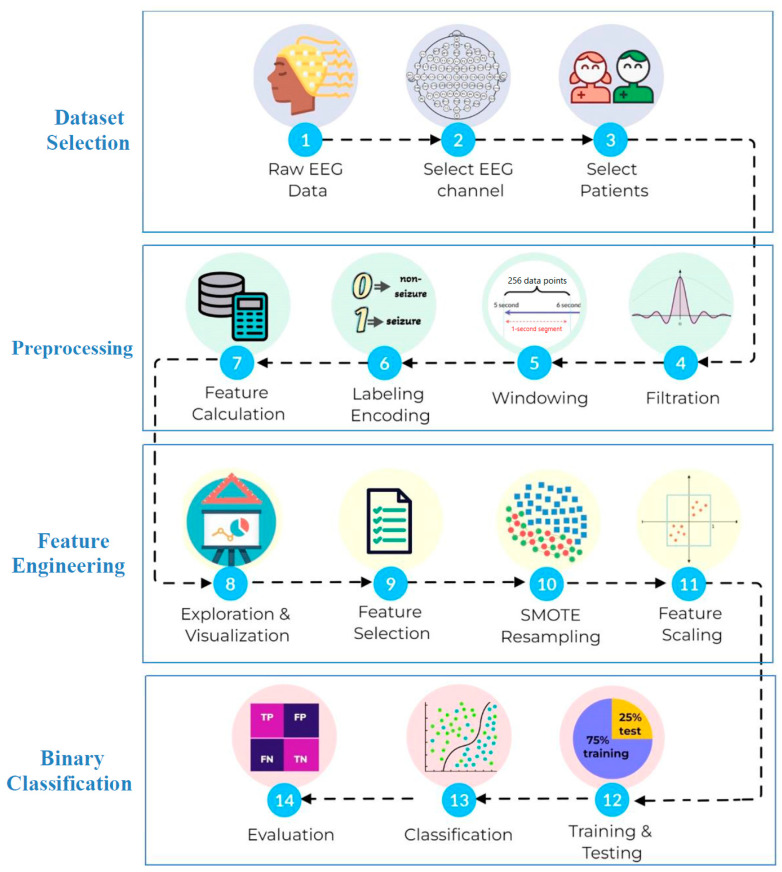
The framework of the development of a binary classifier of seizures.

The framework is explained in detail in Figure 1. The coding in this work was implemented in Python using Jupyter Notebook.

### 2.1. Dataset

Among the available databases of epilepsy recordings, we chose to proceed with the CHB-MIT database. Compared with other databases, CHB-MIT includes the largest number of patients and seizures obtained with scalp EEG, whereas many databases include iEEG and fewer patients and seizure files. It is also widely used, which makes it a good choice for comparing the performance of a proposed classifier to others.

This database was obtained at Children’s Hospital Boston (CHB) in collaboration with a team from the Massachusetts Institute of Technology (MIT). It includes 23 cases collected from 22 epilepsy patients in 2000 and another case added in 2010. Two cases were obtained from the same patient with a 1.5-year duration between the recordings [44].

The database comprises EEG recordings collected noninvasively from 21 channels using a scalp EEG-acquisition cap, with the EEG electrodes positioned according to the international 10–20 system in an anterior–posterior bipolar montage. The positions of the 21 electrodes are Fp1, Fp2, F7, F3, Fz, F4, F8, T7, T8, FT9, FT10, C3, Cz, C4, P7, P3, Pz, P4, P8, O1, and O2. The signals are saved within a total of 664 [.edf] files. The duration of each recording is one hour, with a 256-Hz sampling frequency and 16-bit resolution. The total number of seizures is 198, which are included within 129 files.

To develop an approach suitable for wearable systems, the choice of the EEG channel was based on the cranial location. First, the locations of the electrodes must be adjacent to in – or around the ear electrode placement. Second, the specific channel must connect both sides of the brain. Moreover, considering that temporal lobe seizures are the most common type of focal epileptic seizures [45], EEG electrodes connected to the temporal lobe must be able to sense generalized seizures and most focal seizures. Following these factors, we selected FT9–FT10 as the most suitable channel to achieve these points.

The dataset for training and testing of the classification model was selected from the original CHB-MIT database as shown in Table 1. The selected dataset contains data from twelve patients with ages between 6 and 19 years. The dataset contains all seizure types, including simple partial, complex partial, and Generalized tonic–clonic.

### 2.2. Data Preprocessing

A finite impulse response (FIR) filter between 0.5 and 70 Hz was used to filter the EEG signals [46]. A 1 s rectangular window was applied without overlappingat a sampling frequency of 256 Hz.

Each of these windows was labeled (0) for normal EEG segments, and (1) for seizure segments.

After combining the files of each patient, the files of all patients were combined within one file. Next was features extraction, which is the process of converting raw data into statistical features. Kurtosis, Variance, Energy, and Differential Entropy were calculated from each of the raw segments separately.

Data exploration and visualization were performed to investigate the statistical characteristics of the data, including the range for each feature in each class, the mean, and the standard deviation.

Univariate analysis was performed to study the characteristics of each feature individually, and bivariate analysis was used to study the characteristics of every two features together. By studying the correlation between the features, we found that variance and energy correlate at a rate of 99.99%. Therefore, data cleaning was performed, and variance was deleted to avoid data redundancy.

The features of interest were isolated to form the feature vector to be fed into the proposed classification model as temporal characteristics of the EEG data from FT9-FT10. The used features include the following: (1) Kurtosis, which refers to the statistical measure that describes the shape of any of the tails of a distribution, i.e., whether the distribution is heavy-tailed or light-tailed compared to a normal distribution. (2) Energy, which refers to the capacity to accomplish work. In the context of signals, the energy of a signal over a period of time is the sum of the square of the signal magnitude. (3) Differential Entropy: the “Entropy” of a signal refers to the measure of randomness. Differential entropy is a measure of the average surprise of a random variable for continuous probability distributions. The formulae to calculate these features are listed in Appendix A.

Synthetic Minority Over-Sampling Technique (SMOTE) resampling was used to increase the number of samples of seizures to that of non-seizure segments. Exploring the dataset, we found that seizures made up 0.3% of the complete data. After using SMOTE resampling, we obtained a balanced dataset of 65,400 segments in each of the categories. Feature scaling was utilized to improve the quality of data. This involves normalization and standardization. The primary purpose of normalization is to make the data uniform across all records and fields. It is essential when the data distribution is uncertain or does not have a Gaussian distribution. The technique of putting disparate features on the same scale is known as data standardization. Standardized data can be described as a re-scaling of features so that the mean is 0 and the standard deviation is 1. In this work, the kurtosis and energy data were normalized without assuming a certain distribution of the data, while the differential entropy data were standardized, as their distribution can be assumed to be Gaussian.

### 2.3. Classification

The classification model implemented in this study is the XGBoost approach. The dataset is divided into two subsets for trainingand testing75% of the seizure and non-seizure feature vectors was used for training.

A baseline model with initial parameters was applied to the training dataset to investigate how XGBoost performs in a seizure-detection task based on the extracted features. Then, parameter-tuning was performed to gradually optimize the model to achieve the best results using XGBoost.

To assess the performance of the proposed classification model, several performance measures were computed, including the accuracy, precision, F1 score, recall, false positive rate, and true negative rate, along with a receiver operating characteristic (ROC) curve, the area under the ROC curve (AUC), and the sensitivity of the model to seizures.

## 3. Results

**Figure 2:**
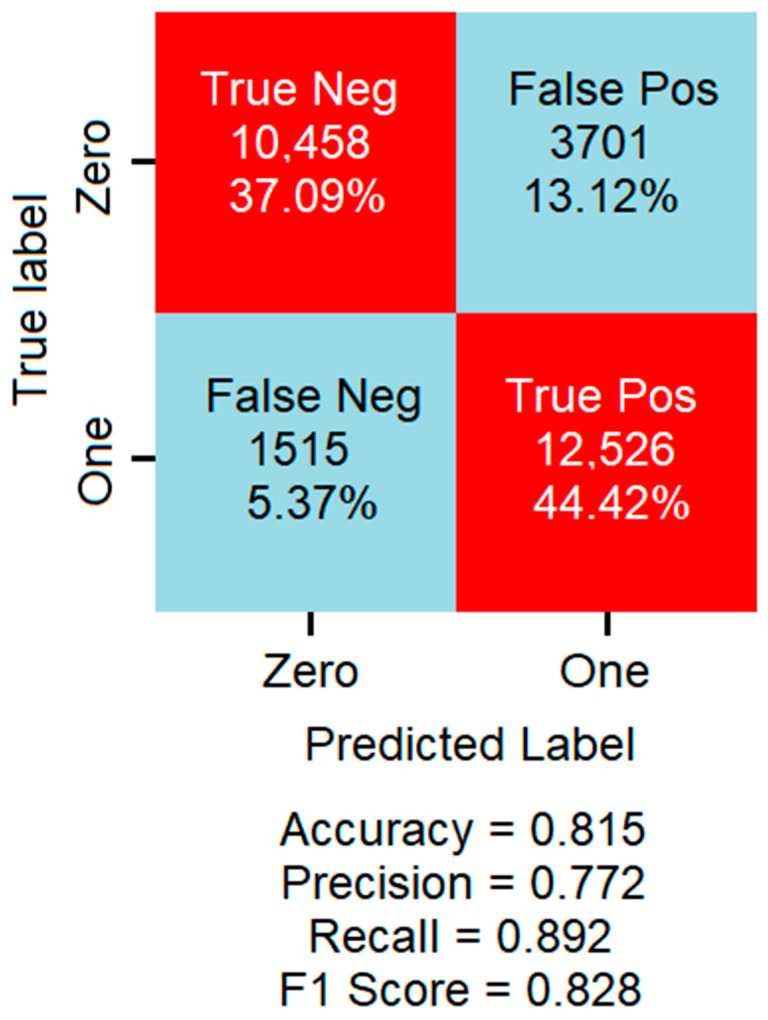
The performance matrix of XGBoost on seizure-detection task.

The performance matrix of XGBoost is shown below in Figure 2. XGBoost achieved a TPR of 89.21%, FNR of 10.79%, a total accuracy of 82%, F1-score of 83%, recall of 89%, specificity of 74%, negative predictive value (NPV) of 87%, positive predictive value (PPV) of 77%, precision of 77%, and error rate of 18.5%

It also achieved a high sensitivity to epileptic events of 89.21% and a tendency of the model to identify and characterize seizures using only three features from only one domain. The FNR is significantly low, but false negatives here may be because not all seizures are visible in all EEG channels; nevertheless, focal seizures can be distinguished from the EEG signal obtained from their associated lobe. Also, generalized seizures do not occur in all brain locations simultaneously, making them observable within one channel at one point in time and not visible in another. On the other hand, the FPR obtained was 26.13%, which may be associated with artifacts. Importantly, the authors who published the database stated that some of the rhythmic activity detected in the EEG was abnormal but not associated with seizures, which can explain the false positives because the classifier is limited to only two classes. In contrast, abnormal segments are associated with a third class that the classifier tends to assign to the class of seizure because it is closer to it than the other class.

**Figure 3:**
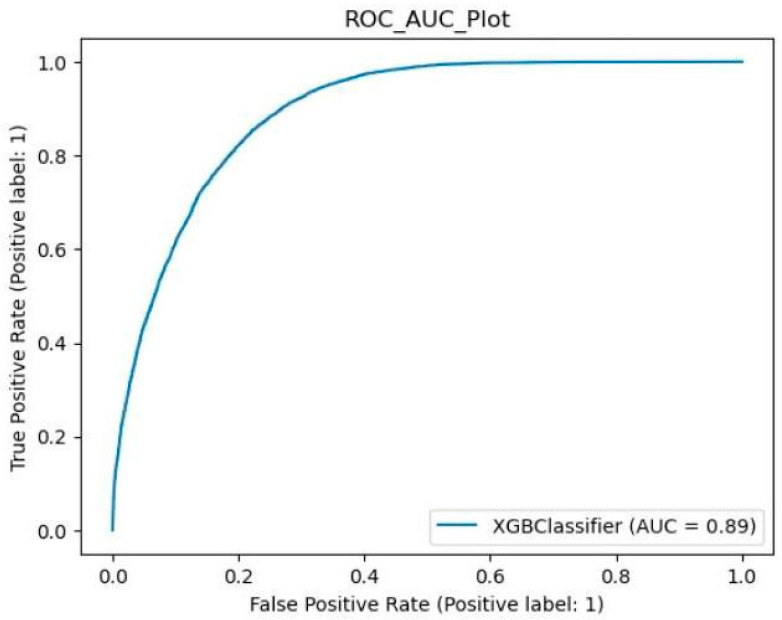
The ROC_AUC plot for XGBoost on seizure-detection task.

The ROC_AUC plot for the XGBoost is shown in Figure 3. The AUC score is 0.89.

## 4. Discussion

Neurologists usually interpret epileptic EEG signals by examining the signal in a time series. In this work, we have used this fact to construct a seizure-classification model based on features taken from the time domain with the simplest possible approach to be implementable in portable devices.

Table 2 presents how the proposed method performs in comparison to the methods in the literature for seizure/non-seizure classification based on the CHB-MIT database as generic approaches (not patient-specific). We can conclude from this aforementioned comparison that the method proposed shows comparable performance represented by the sensitivity achieved while relying on one EEG channel, three features, and a 1 s decision window. It also achieved sensitivity values close to other more complex methods. Hybrid feature vectors obtained from more than one domain achieved higher sensitivity but with the cost of increased complexity and computational cost. Multiple EEG channels may also perform better classification than a single channel, but adding channels leads to reduced patient comfort and increased power consumption. Therefore, the trade-off between simplicity and performance is key to creating the optimal approach for patients with epilepsy.

Considering the instability of the EEG in the case of seizure, the proposed method can be used more effectively for patients with generalized epilepsy and patients with focal epilepsy in the temporal and frontal lobes. Moreover, such an approach could be specified for patients with focal epilepsy within other brain lobes by replacing the EEG channel to be within this lobe and building a model similar to the current model in other respects.

The EEG channels selected in this study are closest to the M1-M2 channels, which are utilized in portable EEG devices using in- or around-the-ear electrodes, which are suitable locations for in-home, self-administered EEG devices. A noteworthy limitation of monitoring seizures using single-channel devices is its sensitivity to seizure type. Several studies have investigated the inclusion criteria and the types/locations of seizures that can reliably be monitored with near-the-ear EEG single-channel monitoring systems, and these studies have been ongoing since the introduction of near-the-ear EEG recording in the early 2000s.

A potential advancement of the presented study is to employ the correlation between sleep and epilepsy [52] and possibly combine both sleep staging and seizure detection. Poor sleep causes a higher potential for seizures to occur and vice versa. In general, there are opposing roles of sleep stages in epilepsy; only 1% or less of seizures arise during rapid eye movement (REM) sleep. In comparison with NREM sleep and wakefulness, REM sleep has an antiepileptic effect against focal and generalized seizures [53]. Besides the difference in the rate of epileptic events throughout the sleep stages, various types of seizures occur during distinct stages [6]. Furthermore, sleep quality may also be affected by medicinal and non-medicinal interventions [54,55].

## 5. Conclusions

There is a demand for continuous monitoring of patients with epilepsy with high performance while maintaining low computational costs suitable for wearable devices. In this work, we show that XGBoost can be used to provide a classification of seizures based on a few temporal features obtained from a single EEG channel, with the aim of building a simple ML algorithm for use in wearable systems.

The FT9–FT10 channel was chosen so that the signal comes from the area as close as possible to the ear to utilize in- or around-the-ear-based recording systems. In contrast to features computed from the frequency domain and others, energy, differential entropy, and kurtosis were chosen to represent the signal as features computed from the time domain, such as those of raw EEG, provided that no additional calculations were required. Finally, XGBoost has been proposed as a classification model because it provides high performance in such tasks quickly and with low computation.

The results presented after testing the proposed model on the test dataset show that XGBoost is effective in detecting seizures, with high sensitivity, which makes it a candidate for AI software in wearable devices for epilepsy patients. In addition, we concluded that features from the time domain alone can be used to distinguish seizures and that seizures can be observed in the signal obtained from the near-ear EEG channel. Moreover, we have shown with the results that the use of a 1 s EEG segment is sufficient to obtain the information needed for seizure detection and is also important for real-time application. Computed from the time domain alone for just a fraction of a second, and based on a simple ML model, we can use these data as input to achieve a high seizure sensitivity of up to 89%.

This study aimed to develop a simple yet reliable algorithm to automate seizure-classification tasks, which can then be implemented in existing portable recording devices. The results have indicated that the classification performance of the proposed algorithm is comparable with more advanced approaches while retaining simplicity and low computational costs. A continuation of this work can focus on performing comparison studies of seizure monitoring and classification between standard clinical EEG monitoring and a portable single-channel EEG monitoring device that implements the proposed algorithm.

## Figures and Tables

**Table 1:** Details of the dataset from MIT-CHB database included in the study.

No.	Patient	Gender	Age	Seizures (s)
1	chb-02	M	11	184
2	chb-04	M	22	400
3	chb-05	F	7	524
4	chb-07	F	14.5	340
5	chb-09	F	10	296
6	chb-11	F	12	816
7	chb-14	F	9	177
8	chb-15	M	16	2012
9	chb-16	F	7	77
10	chb-17	F	12	296
11	chb-19	F	19	239
12	chb-20	F	6	302

**Table 2:** Comparison between the proposed method and state-of-the-art methods for seizure classification.

Reference	Channel Count	Window Length	Features (Input Shape)	Feature Domain	Classification	Sensitivity
Zabihi 2016 [47]	23	1 s	7 features	Non-Linear	LDA and NB	88.27%
Sopic 2018 [48]	2	4 s	7 features	Nonlinear and Power	Random Forest	93.80%
Wei 2019 [49]	23	5 s	Waveform image (1280 × 23 × 1)	Time	CNN	72.11%
Ayodele 2020 [36]	8	5 s	17 10-layer 16 × 16 raster arrays	Frequency	RNN	71.45%
Liang 2020 [50]	18	2 s	waveform image (100 × 227 × 1)	Time	LRCN	84.00%
Hu 2020 [51]	23	4 s	10 features	Non-Linear	Bi-LSTM	93.61%
Proposed Approach	1	1 s	3 features	Time	XGBoost	89.21%

## Data Availability

The used database is available online at PhysioNet (https://physionet.org/physiobank/database/chbmit/). Accessed on 13 January 2023.

## Appendix A. Features Formulae

$$\mathrm{Kurtosi}=n\times \frac{\sum_{i}^{n}{\left(Y_{i}-\bar{Y}\right)}^{4}}{\sum_{i}^{n}{\left(Y_{i}-{\bar{Y}}^{2}\right)}^{2}}$$

where the following apply:

$Y_{i}$: the *i*th Variable of the distribution.

$\bar{Y}$: Mean of the distribution.

n: No. of variables in the distribution.

$$\mathrm{Energy}=\sum_{n=-\infty }^{\infty }{\left|x[n]\right|}^{2}$$

where *x*[*n*] is a discrete-time signal.

$$\mathrm{Differential}\;\mathrm{Entropy}\;(\Delta \mathrm{Entropy})=\int_{-\infty }^{+\infty }p\left(x\right)\;{log}_{2}p\left(x\right)\;dx$$

where *p*(*x*) is the Probability Density Function.
